# A review of specialist palliative care provision and access across London – mapping the capital

**DOI:** 10.1080/17571472.2016.1256045

**Published:** 2016-12-26

**Authors:** Sarah Cox, Fliss E. M. Murtagh, Adrian Tookman, Andrew Gage, Nigel Sykes, Maureen McGinn, Meeta Kathoria, Hilary Wilderspin, Liz Chart

**Affiliations:** a RM Partners, London, UK; b Chelsea and Westminster NHS Foundation Trust, London, UK; c London Cancer Alliance, London, UK; d King’s College London, London, UK; e PallE8, London, UK; f Marie Curie, London, UK

**Keywords:** Using MeSH headings, data collection, hospice care, hospices, London, palliative care

## Abstract

Palliative care provision varies by diagnosis, geography, and setting. The Minimum Data-set provides high-level data on provision, but comprehensive comparative information about specialist palliative care (SPC) provision is lacking. The London Cancer Alliance – now RM Partners’ Accountable Cancer Network - palliative care group (West/South London) and PallE8 (North/East London), with Marie Curie, sought to address this gap. The aim was to provide comparative data on SPC provision across London to support commissioners and providers to assess provision, identify gaps, and reduce inequity. A data-collection template was developed through expert consensus. Demographic, diagnostic, and service data was collected, plus models of care, staffing levels, and use of clinical outcome/experience measures. Results were collated by organisation and CCG. Cleaned data was provided back to each organisation for verification before final analyses.

**Results:**

All 50 adult SPC providers in London participated, representing hospitals, hospices and community services.

• Patients in all 32 CCGs have access to hospice beds, with 322 beds from 15 providers (4 NHS) for a population of 9,323,570 (with 47,583 deaths annually).
• SPC in London sees more non-cancer patients than is reported nationally; 79% of hospital advisory, 74% of community, and 88% of hospice in-patient services have higher proportions of non-cancer patients.
• Considerable variation in out-of-hours availability of both hospital SPC and community SPC services across London; only 9 of 30 hospital and 17 of 26 community services provide seven-day visiting.
• Wide variation in the models of community-based SPC; proportions of community patients attending day services vary from 1 in 4, to 1 in 17, just 13 CCGs have H@H-type provision, with few Rapid Response or Care Coordination services.

**Conclusions:**

This detailed survey demonstrates important gaps in availability and provision of SPC services. Recommendations are made for commissioners and providers to join together to address these. It also gives a comprehensive view of rapidly changing models of community-based care, to inform innovation and service development.

## Why this matters to me

Modern health services and teams operate in silos with organisations having great choice over the services they develop. This has advantages in terms of being able to develop locally relevant services but may also result in inequality and inequity in services geographically. London has a very diverse population but advanced progressive diseases occur across the capital and specialist palliative care services should be available to all Londoners. This report arose from our curiosity to map formally what we had observed and to provide services and commissioners with objective information about specialist palliative care in their area.

## Key messages

• There are important gaps in Specialist Palliative Care service availability and provision across London
• The out of hours service availability of SPC does not meet NICE minimum standards from 2004
• SPC services see mostly cancer patients and there may be a large degree of unmet need for SPC amongst the non-cancer population

## Introduction

Specialist palliative care is defined as

the active, total care of patients with progressive, advanced disease and their families. Care is provided by a multi-professional team who have undergone recognised specialist palliative care training. The aim of the care is to provide physical, psychological, social and spiritual support … [1]

**Figure 1.**
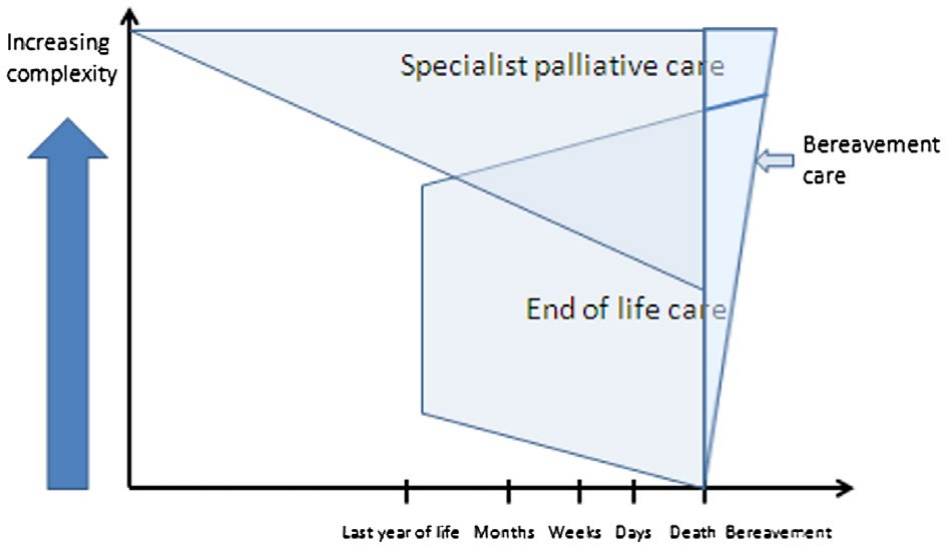
Relationship between specialist palliative care (SPC) and end of life care.

Specialist Palliative Care (SPC) can be valuable to support patients at the end of life but is also important earlier on in the disease process (Figure 1). Earlier referral to palliative care services has been associated with financial savings and even survival benefits of up to three months.[2–4]

In 2013/2014 the first pan-London mapping of SPC services was carried out across London. The aim was to understand more about SPC provision in London with a view to making specific recommendations to providers and commissioners to benefit patients and those close to them. Data was collected about numbers of patients seen over the previous 12 months, their diagnosis and the types and availability of SPC services.

The SPC needs of patients with non- cancer diagnoses such as COPD and heart failure are similar to those of patients with cancer.[5–8] We should expect that the population seen by SPC services should reflect the populations that die with these conditions. ONS figures show that approximately 29% patients died from cancer in England and Wales in 2013 [9] but cancer patients make up 70–80% of patients seen by specialist palliative care services [10].

The service mapping would compare service availability with NICE guidance 2004 [11] which set out minimum standards for availability of specialist palliative care services;

The team should be staffed to a level sufficient to undertake face-to-face assessments of all people with cancer at home or in hospital, 09.00–17.00, seven days a week. In addition, there should be access to telephone advice at all times (24 h, seven days a week). This is considered a minimum level of service.

The data collection did not set out to measure the quality of care provided by SPC services across London. Indeed there has not been an accepted measure for quality nationally, although this is being addressed by a PHE project reporting in April 2016. This aims to recommend measures for SPC services that can be used to assess the outcome of SPC intervention, benchmark services and improve quality of care for patients and those close to them.

There is some information about the quality of end of life care services in London which suggests there is room for improvement. The national bereavement survey (VOICES, 2012) placed London lowest of all regions in England on an overall measure of quality of end of life care [12].

## Methods

The service mapping reported on in this paper was based on previous data collections in different parts of London. However, the data had not previously been put together so that comparisons could be made across the capital. This data collection reflects collaboration between the two umbrella organisations for SPC services in London (one a part of the London Cancer Alliance – now RM Partners’ Accountable Cancer Network – and PallE8) and Marie Curie.

A template was designed and refined through expert consensus and previous use. The template expanded on the established National Council for Palliative Care minimum data-set to collect not only essential numerical, demographic and diagnostic information for patients seen over the most recent 12-month period, but also the types and availability of SPC services provided across London. Information was also collected on providers’ staffing levels at one specified time point within the year and their use of clinical outcome measures.

Patient level data was not collected. This does introduce the possibility of double counting of patients by different services.

The template was completed by all 50 adult SPC providers in London, as well as by paediatric SPC services in North Central and North East London, and covered services in hospitals, hospices and the community. The results were correlated, both by each organisation and by the 32 CCGs in the Capitol. Data was checked and cleaned, with detailed checking with providers for any missing or obviously incorrect data (including outliers), then provided back to each organisation in report format for final checking before being accepted as correct.

## Results

### Types of SPC services provided

#### Inpatient hospices

Patients in all of London’s 32 CCGs have access to in-patient hospice beds. There are a total of 322 hospice beds for a population of 9,323,570 (with 47,583 deaths annually). These are provided by 15 organisations of which only four belong to the NHS, the remainder being charitably owned and largely charitably funded.

#### Hospital SPC teams

All the multi-speciality hospitals serving London have palliative care teams, and in all but one case, are funded directly by the NHS Trust concerned. The specialist centres, including The Royal Marsden, The Royal Brompton and Harefield, and Queen Square (this centre is covered by the CNWL UCLH SPC service) also have palliative care teams.

#### Community SPC teams

Community specialist palliative care refers to teams of palliative care clinical nurse specialists with palliative medicine specialist support. They can visit patients in their own homes as well as provide telephone advice. The results show that all London CCGs have access to this type of service during weekday working hours; however, the facilities available at other times vary considerably between providers.

#### Day therapy

Palliative care day therapy services provide a range of creative and rehabilitation activities for community patients as well as the opportunity to maintain and renew social interactions. Day care is available in most CCGs across London but exceptions exist, e.g. in Waltham Forest. The numbers attending day services as a proportion of those receiving community SPC vary substantially, e.g. about one in 17 in Hounslow, one in 10 in West London, one in eight in Newham and one in four in Bromley.

#### Outpatient clinics

Palliative care services may provide outpatient clinic facilities for patients fit enough to travel, sometimes in response to the need for a specific professional intervention, e.g. from a doctor or a social worker, or, alternatively, as a potentially more efficient use of nursing resources than making a home visit the basis of every face-to-face encounter. SPC outpatient facilities are provided in all but five of London CCG areas (Hounslow, Merton, Sutton, Haringey and Islington) but the number of patients involved tends to be small compared with those receiving community SPC.

#### Hospice at home

Hospice at Home (H@H) provides extra hands-on nursing care to complement the statutory district nursing service and the usual advisory role of the palliative care clinical nurse specialist. H@H-type services exist in 13 CCGs from one or more of seven providers. Some of these services cater only for patients already known to the provider’s usual community SPC team while others receive referrals directly. There is a large variation in the ratio of patients receiving H@H input to the total number of patients receiving community SPC, from around 1:25 in Richmond, to 1:6 (a fairly typical figure) in Harrow, to 1:1.5 in Greenwich.

A small number of CCGs have SPC-run Rapid Response or Care Coordination services.

### SPC providers and CCG boundaries

The data indicates that there are numerous instances in which the same type of service for a single CCG is split between two or more providers. The reason for this in the case of hospital palliative care teams is clear. According to specialty and sometimes locality, a CCG’s residents are likely to enter different hospitals and their palliative care needs during an admission are dealt with by the SPC team of the hospital involved. In relation to community services, the reason for multiple providers is largely historic. Areas served by particular community SPC teams were delineated under a previous phase of NHS organisation and, indeed, were often separate from it. They therefore have boundaries that often do not match those of today’s CCGs. This can also apply to the catchment areas of hospice in-patient units, which are likely to extend across all or parts of more than one CCG.

Of the 32 London CCGs, 19 have a single provider for SPC in-patient (i.e. hospice) services and 13 have a single provider for community SPC. For 12 CCGs, each type of service is provided by a single provider and, in 11 cases, this is the same provider for both service types. Other CCGs have up to four community SPC providers (e.g. Ealing, Camden) and three hospice in-patient providers (e.g. Islington, West London).

### SPC Service availability

Hospice in-patient SPC services all provide face-to-face support seven days a week at all hours.

For *hospital SPC services* across London;

• Only 9 of 30 services were able to provide seven-day visiting services.
• Four services do not provide telephone advice out of hours (Princess Alexandra Hospital, Barnet and Chase Farm Hospital Trust, North Middlesex Hospital and Whittington Hospital).
• Three services are providing a six-day visiting service.
• However, six services are providing face-to-face visiting all hours, which represents best practice (University Hospital Lewisham; King’s College Hospital NHS Foundation Trust; Central and North West London – University College London Hospitals service; Central and North West London HCA Specialist Palliative Care Service; Royal Marsden NHS Foundation Trust; and Guy’s and St Thomas’ NHS Foundation Trust).

For *Community SPC services* across London;

• 17 of 26 services are providing seven-day visiting.
• Five services are unable to provide telephone advice to professionals out of hours (St Clare Hospice, Royal Free Hospital, Haringey Community Team, Diana Team Newham [paediatric palliative care] and North East London NHS Foundation Trust’s Redbridge Specialist Palliative Care Team).
• Six services are unable to provide telephone advice to patients or their families out of hours (as above, with the addition of University Hospital Lewisham).
• However, five services demonstrate best practice by providing face-to-face visiting at all hours (Saint Francis Hospice; Central and North West London – Camden; Central and North West London – Islington ELiPSe; Guy’s and St Thomas’ NHS Foundation Trust; and St Christopher’s Hospice).
• There is a large variation in service availability of community SPC between CCGs, with some CCGs providing 24/7 specialist care visiting and others providing only Monday to Friday, 9am–5 pm services.
• Some variation of service availability exists within CCGs as a result of CCGs having more than one provider, with some CCG residents receiving significantly greater service than others

Since this service mapping exercise, the authors are aware of some further services which have been able to implement seven-day, face-to-face visiting. Despite this, the data reveals that SPC service availability across London is still below the minimum service level set by NICE in 2004.[11]

### Cancer/non-cancer diagnosis of SPC patient population

#### Hospital advisory teams

The percentage of SPC patients seen with non-cancer diagnoses varied from 15 to 50% excluding specialist hospitals seeing exclusively cancer or exclusively no-cancer patients.

• 22 out of 28 (79%) services have a higher non-cancer patient rate compared to the national average for hospital advisory teams which is 25%.[10]

#### Hospice in-patient units

• 2014 data indicate that, of the patients seen by in-patient units, the percentage with non-cancer diagnoses varied from 5 to 30%.
• 14 out of 16 units (88%) have increased their non-malignant referral proportion of non-cancer patients to adult in-patient units to a level higher than the national average.[10]

#### Community palliative care

• 2014 data indicate that, of the patients seen by community palliative care teams, the percentage with non-cancer dsiagnoses varied from 10 to 35%.
• The study data indicates that 17 out of 23 (74%) adult SPC services in London saw more patients with non-malignant illnesses than the national figure.[10]

## Discussion

This was the first time London wide data has been collected about SPC services. It demonstrates that SPC services are available across London at home, in hospital and in hospices in weekday working hours. However, SPC out-of-hours service availability falls short of national guidance and accepted best practice.

London SPC services see a disproportionate number of people with cancer compared to deaths from cancer, although the data we have analysed show that London SPC services are seeing more patients with non-malignant disease than the national average.[10]

The analysis of this data is limited by the fact that we were not able to collect patient level data. We are also unable to report on quality outcome measures outside service availability and diagnoses as there are no nationally accepted measures of SPC outcomes or service quality to act as benchmarks. The anticipated launch of a national individual-level data-set in 2017 of SPC services including demographic details, activity information and patient outcomes data will be an important milestone towards providing evidence on outcomes, and (in the longer term) facilitating genuine equity of access across London.

Given that demands on existing stretched resources are only set to grow, we must tackle these issues and find solutions to avoid failing vulnerable people across London at the time they need us most. Monitoring the changing provision of SPC services across London is an important step along the road to improving care.

## Disclosure statement

No potential conflict of interest was reported by the authors.

## Ethical committee approval

The Palliative Care Group of the London Cancer Alliance – now RM Partners’ Accountable Cancer Network, hosted by the Royal Marsden NHS Foundation Trust - oversaw this work.
